# A Novel Hybrid Cytokine IL233 Mediates regeneration following Doxorubicin-Induced Nephrotoxic Injury

**DOI:** 10.1038/s41598-019-39886-9

**Published:** 2019-03-01

**Authors:** Vikram Sabapathy, Nardos Tesfaye Cheru, Rebecca Corey, Saleh Mohammad, Rahul Sharma

**Affiliations:** Center for Immunity, Inflammation and Regenerative Medicine (CIIR), Division of Nephrology, Department of Medicine, University of Virginia, PO Box 800133, Charlottesville, VA 22903 USA

## Abstract

Kidney injury, whether due to ischemic insults or chemotherapeutic agents, is exacerbated by inflammation, whereas Tregs are protective. We recently showed that IL-2 and IL-33, especially as a hybrid cytokine (IL233 - bearing IL-2 and IL-33 activities in one molecule), potentiated Tregs and group 2 innate lymphoid cells (ILC2) to prevent renal injury. Recent studies have indicated a reparative function for Tregs and ILC2. Here, using doxorubicin-induced nephrotoxic renal injury model, we investigated whether IL233 administration either before, late or very late after renal injury can restore kidney structure and function. We found that IL233 treatment even 2-weeks post-doxorubicin completely restored kidney function accompanied with an increase Treg and ILC2 in lymphoid and renal compartments, augmented anti-inflammatory cytokines and attenuated proinflammatory cytokine levels. IL233 treated mice had reduced inflammation, kidney injury (Score values - saline: 3.34 ± 0.334; IL233 pre: 0.42 ± 0.162; IL233 24 hrs: 1.34 ± 0.43; IL233 1 week: 1.2 ± 0.41; IL233 2 week: 0.47 ± 0.37; IL233 24 hrs + PC61: 3.5 ± 0.74) and fibrosis in all treatment regimen as compared to saline controls. Importantly, mice treated with IL233 displayed a reparative program in the kidneys, as evidenced by increased expression of genes for renal progenitor-cells and nephron segments. Our findings present the first evidence of an immunoregulatory cytokine, IL233, which could be a potent therapeutic strategy that augments Treg and ILC2 to not only inhibit renal injury, but also promote regeneration.

## Introduction

Kidney Injury is a global health problem associated with high healthcare costs, mortality, and morbidity^1^. Other than the dialysis, no other therapeutic intervention has been shown to increase survival. The immune system plays a vital role in both worsening as well as ameliorating the kidney dysfunction. Understanding the convoluted interplay between the immune cells and tissue resident cells could help us to understand the complex pathophysiological outcome during the injury and repair process. Previous studies have demonstrated that CD4^+^Foxp3^+^ Regulatory T Cells (Tregs) play a vital role in suppressing inflammation and protecting renal function^2–5^ using both cytokine-independent and cytokine-dependent pathways^3,6^. While exogenous Treg therapy is promising, the scale-up of Treg-production for clinical applications presents challenges such as heterogeneity during *in vitro* expansion, cost, dosing, number of injections as well as stability and longevity of the transplanted cell are among the issues that need to be resolved^7^. Hence, the strategy of using cytokines such as IL-2 for expansion of endogenous Tregs could be more efficient and cost effective^8,9^.

The recently identified group 2 innate lymphoid cells (ILC2) is an innate immune cell subset derived from common lymphoid progenitors. ILC2 have been shown to exhibit functions analogous to Th2 cells and have been broadly implicated in contributing to tissue development and homeostasis^10^. ILC2 are identified by the constitutive expression of ST2 (IL-33 receptor). IL-33 has been shown to strongly induce the activation of ILC2 and promote cytokine secretion^10^. ILC2 also express high-affinity IL-2 receptor and IL-2 induces proliferation of ILC2^11^.

ILC2 and Treg have been recently shown to facilitate repair of lung epithelium following injury^12,13^. Our studies have recently demonstrated that Tregs express IL-33 receptor (ST2) and that IL-2 and IL-33 synergize to promote the expansion of Tregs and ILC2. Interestingly, a hybrid cytokine – IL233, which contains IL-2 and IL-33 activities in a single molecule was more efficient than the mixture of IL-2 and IL-33 in expanding Tregs and protecting mice from ischemia reperfusion injury (IRI)^14^. Importantly, treatment with IL233 within hours after renal IRI prevents inflammation, injury, and mortality^14^. We hypothesize that treatment with IL233 hybrid cytokine, which results in stimulation and expansion of Tregs and ILC2 to prevent inflammation, may also contribute towards regeneration.

Renal dysfunction is a major source of concern in the use of chemotherapeutic agents^15^. The prevalence of nephropathy in patients suffering from cancer is over 60%^16,17^. Doxorubicin (trade names Adriamycin, Rubex) is an anthracycline class of drugs employed for more than 30 years to treat cancers^18^. Doxorubicin is a potent anti-cancer drug, however, the acute and chronic toxic side effects remains a major concern in its usage^18^. Although doxorubicin is known more for cardiotoxicity in humans and less for nephrotoxicity, it causes severe nephrotoxicity in rodents in a very predictable manner with high penetrance^19,20^. For this reason, doxorubicin-nephrotoxicity has been used as a reliable and reproducible model of renal injury to study pathology and therapeutic strategies^19^.

In this study, we examined the efficacy of IL233 in facilitating recovery of the murine kidney from nephrotoxicity, with intervention at early, late or very late time points after doxorubicin administration. The structure and function of kidneys were probed using flow cytometry, histology, quantitative gene expression analysis, immunohistochemistry, and biochemical assays.

## Results

### IL233 treatment after doxorubicin administration preserves renal function

**Figure 1:**
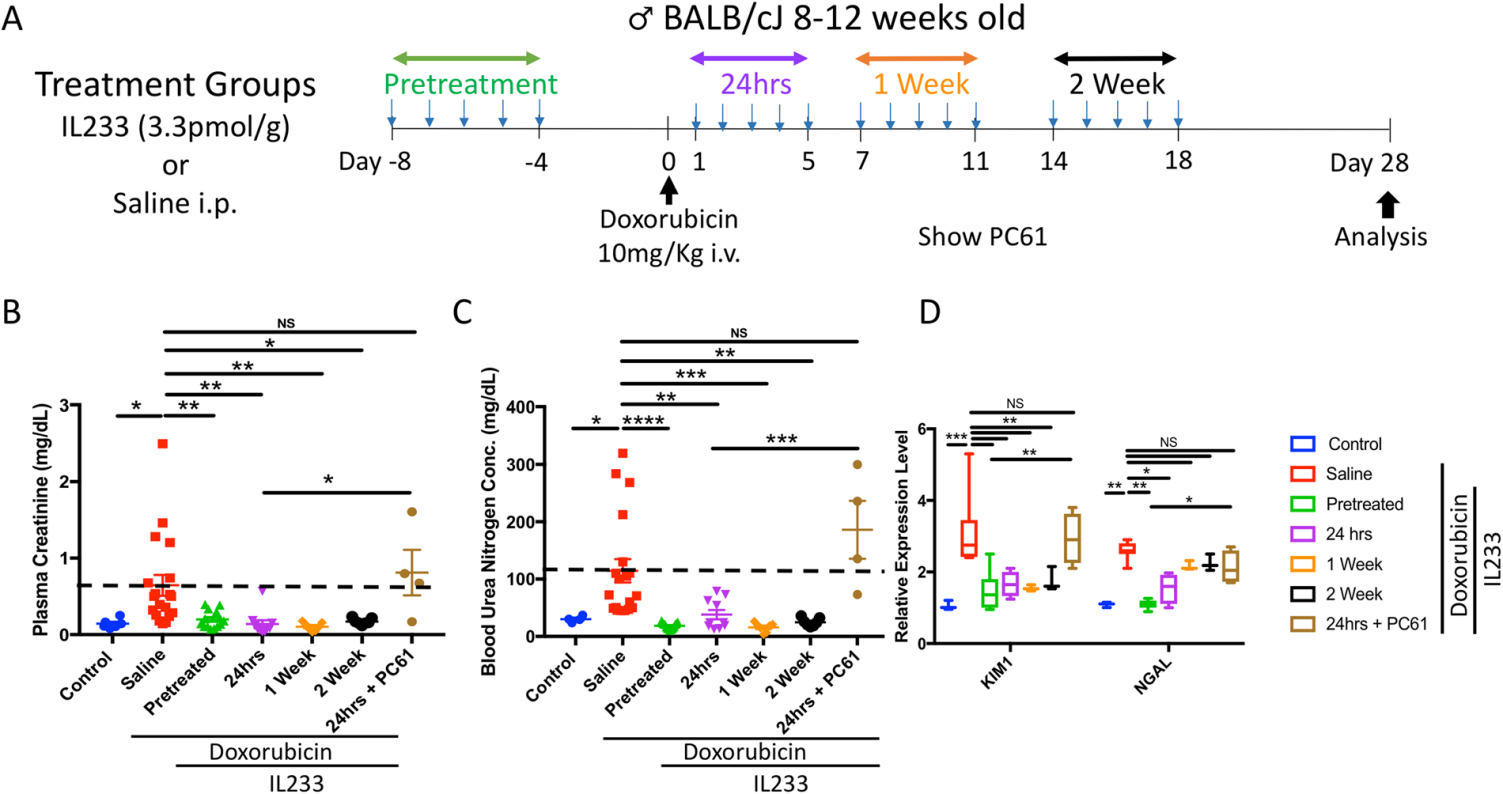
IL233 treatment both before and after doxorubicin administration preserves renal function. (**A**) Experimental Design; Mice were injected with saline or IL233 for 5 consecutive days either 8 days before (pretreatment) or starting 24hrs, 1 week or 2 weeks after doxorubicin injection. (**B**) Plasma Creatinine, day 28; (**C**) Blood Urea Nitrogen, day 28; (**D**) Real-time PCR analysis of kidney injury markers *Kim1* and *Ngal*, day 28, n ≥ 5. The dotted lines represent the mean of the control group. Symbols represent individual mice; mean ± SEM is shown. * p < 0.05; **p < 0.01; ***p < 0.001; ****p < 0.0001; NS p > 0.05 by one-way ANOVA.

Kidney injury was induced in the mice by intravenous (i.v.) administration of doxorubicin. The animals were divided into different treatment groups. The groups were control (no treatment), pretreatment (injection of IL233 daily for 5 consecutive days before administering doxorubicin), 24 hours (IL233 injection daily for 5 days starting 24 hours after doxorubicin), 1week (IL233 injection daily for 5 days starting 1 week after doxorubicin) or 2 week (IL233 injection daily for 5 days starting 2 weeks after doxorubicin). The saline control mice received 5 injections of saline instead of IL233 (Fig. 1A). Please see the supplementary information for details of the number of animals in each experiment. The mice were euthanized 4 weeks after doxorubicin administration for further analysis. To determine the kidney function we measured plasma creatinine (PCr) (Fig. 1B) and blood urea nitrogen (BUN) levels (Fig. 1C). Based on the levels of PCr and BUN, the renal function was significantly protected in the cytokine treated mice when compared to the saline group. Interestingly, not only pretreatment but IL233 treatment as late as 2 weeks post-doxorubicin administration rescued the function of the kidney to the levels of control naïve mice. In order to assess renal injury, we performed quantitative gene expression analysis of markers of renal injury, kidney injury molecule-1 (*Kim-1*) and neutrophil gelatinase-associated lipocalin (*Ngal*) (Fig. 1D), which indicated that the degree of kidney injury was very high in saline-treated mice as compared to all the cytokine treated groups. There was no difference in the level of *Kim-1* as compared to the naïve control mice among the treated groups; however, the level of *Ngal* was higher in the post-treatment groups as compared to the pretreatment and untreated control groups. To examine whether the protection was related to Tregs, a subset of mice treated with IL233 cytokine 24 hours after doxorubicin administration were also injected with the Treg-depleting antibody to IL-2Rα (PC61), which resulted in severe kidney dysfunction as suggest by plasma creatinine, blood urea nitrogen and *Kim-1* and *Ngal* gene expression analysis.

### IL233 treatment increases the number and proportion of Tregs and ILC2

**Figure 2:**
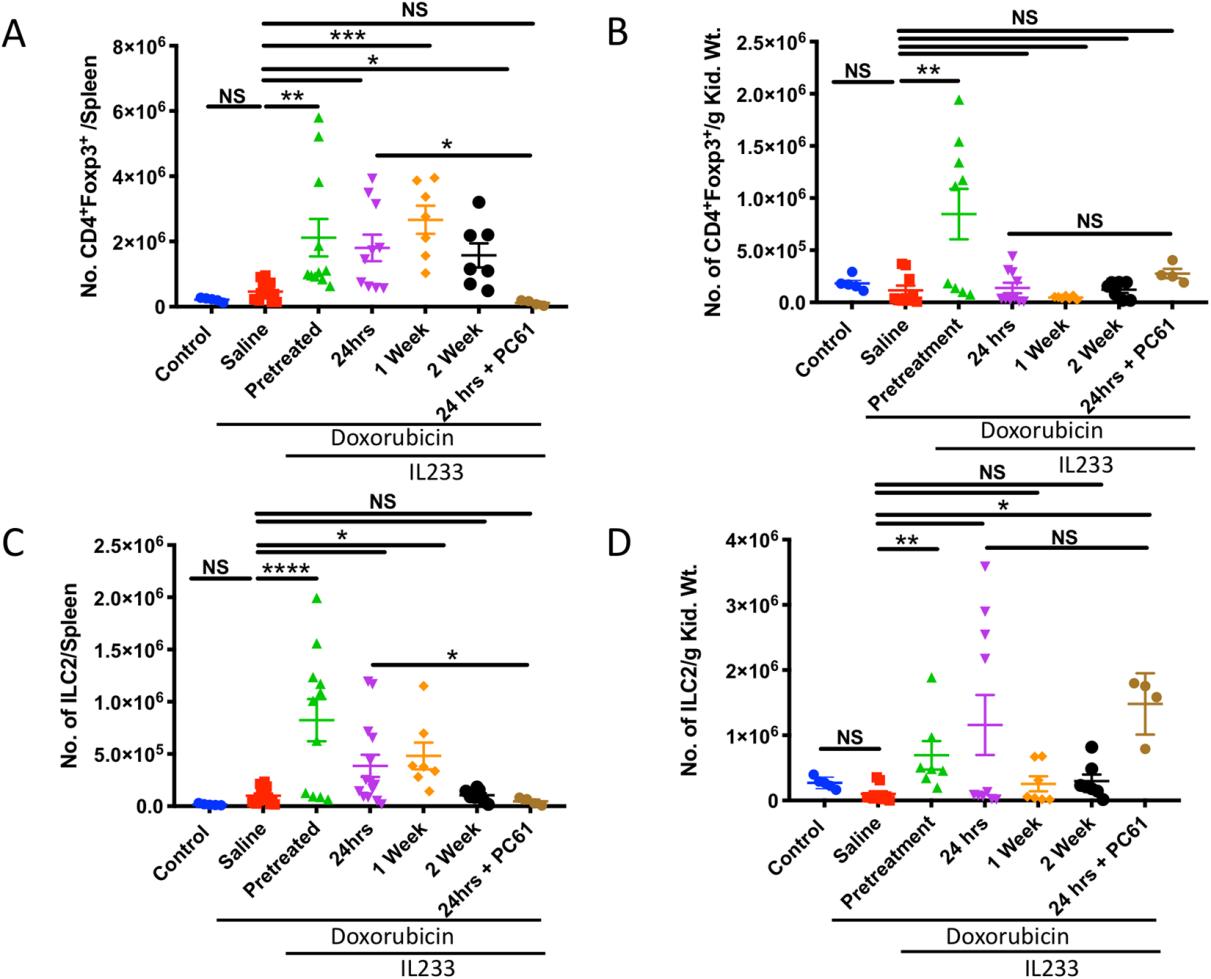
IL233 treated mice had a higher proportion of Tregs in spleen and kidney. The proportion of Tregs, identified as CD4^+^Foxp3^+^ cells is shown in (**A**) Spleen, (**B**) Kidney; Proportion of ILC2, identified as Lin^−^ST2^+^CD90^+^ in (**C**) Spleen, (**D**) Kidney. Symbols represent individual mice; mean ± SEM is shown. *p < 0.05; **p < 0.01; ***p < 0.001; ****p < 0.0001 NS p > 0.05 by one-way ANOVA.

**Figure 3:**
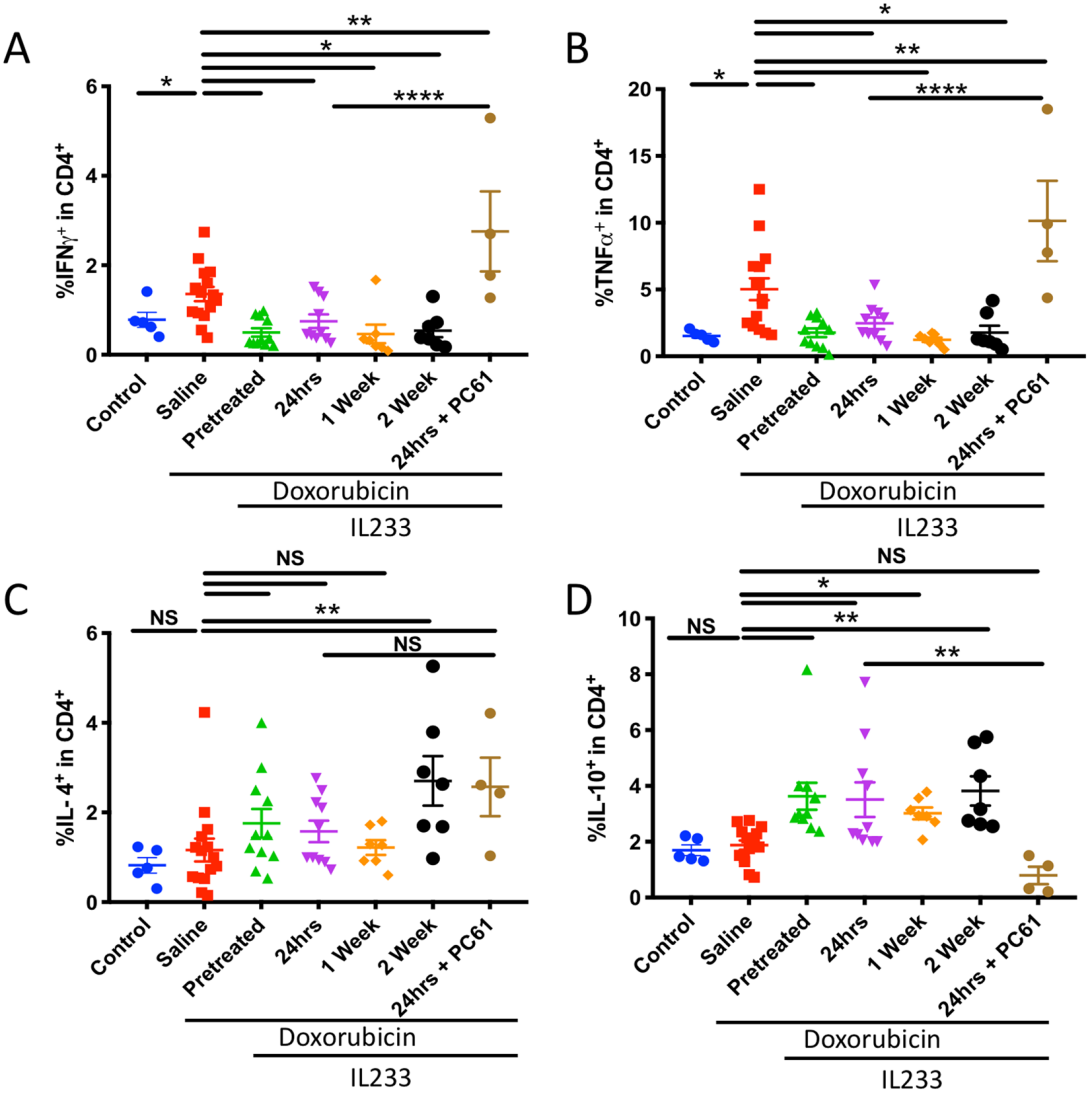
IL233 attenuates pro-inflammatory cytokines and augments anti-inflammatory cytokines. Pro-inflammatory cytokines (**A**) IFNγ^+^CD4^+^, (**B**) TNFα^+^CD4^+^; or anti-inflammatory cytokines (**C**) IL-4^+^CD4^+^, (**D**) IL-10^+^CD4^+^. Symbols represent individual mice; mean ± SEM is shown. *p < 0.05; **p < 0.01; ****p < 0.0001; NS p > 0.05 by one-way ANOVA.

Flow cytometry analysis of spleen and kidney revealed that the numbers of Tregs (Fig. 2A,B) were, in general, higher in the IL233 treated groups as compared to saline. The level of Tregs was also significantly elevated in the kidneys of mice pretreated with IL233. Mean Treg levels, though elevated, did not reach statistical significance in other treatment groups despite the protection from renal injury and dysfunction (Fig. 2B). Similarly, the number of ILC2 was significantly higher in the spleens of pretreated, 24 hours and 1-week post-treated groups compared to saline (Fig. 2C). The ILC2 level in the kidneys was also significantly higher in the pretreatment group but did not reach statistical significance in the other treatment groups although the mean ILC2 levels were higher than the saline controls (Fig. 2D). The data suggest that Tregs and ILC2 levels increase in the spleen and kidney, may suppress inflammation and participate in repair. To determine the role of Tregs, mice were injected with anti-CD25 mouse monoclonal antibody (PC61) in the 24 post-treatment group. The results indicate that Treg numbers in spleen were significantly reduced upon PC61 administration, however, no significant reduction of Tregs was observed in the kidneys. A similar trend was also observed for ILC2, which also express CD25^11^. PC61 administration reduced the number of ILC2 in the spleen but not in the kidneys. The data suggest that although PC61 treatment did not show a statistically significant reduction in the numbers of Treg and ILC2 in kidneys (see Fig. 3 below), the function of the these Tregs could be compromised leading to severe impairment of kidney function. It couldn’t be determined whether the Tregs in kidneys may either be tissue resident Tregs or could be rapidly repopulated from lymphoid organs, once the effect of PC61 waned.

### Treatment with IL233 augments anti-inflammatory cytokines and attenuates pro-inflammatory cytokines

We evaluated the pro- and anti-inflammatory cytokine production by the T-helper (Th) cells in the mice treated with IL233. Single cell suspension of spleen cells was stimulated with PMA (Phorbol 12-Myristate 13-Acetate) and ionomycin, in the presence of monensin for five hours and analyzed by flow cytometry for intracellular cytokine expression. Treatment with IL233 attenuated the expression of the pro-inflammatory cytokines IFNγ (Fig. 3A) and TNFα (Fig. 3B). On the other hand, the levels of Th2 cytokine IL-4 (Fig. 3C) and anti-inflammatory cytokine IL-10 (Fig. 3D) were considerably elevated in all treatment groups, with the pretreatment and 2-week post-doxorubicin groups reaching statistical significance as compared to the saline group. Between the IL233-treated groups, pre-treatment, as well as post-treatment strategy, exerted better anti-inflammatory response whereas pro-inflammatory cytokine levels were very high in saline-treated group. The group injected with PC61 antibody exhibited higher levels of pro-inflammatory cytokine and levels of anti-inflammatory cytokine IL-10 was highly attenuated, however, PC61 treatment did not affect IL-4 production though the mean values were higher than the corresponding group without PC61.

### Reduced kidney injury in IL233-treated mice

**Figure 4:**
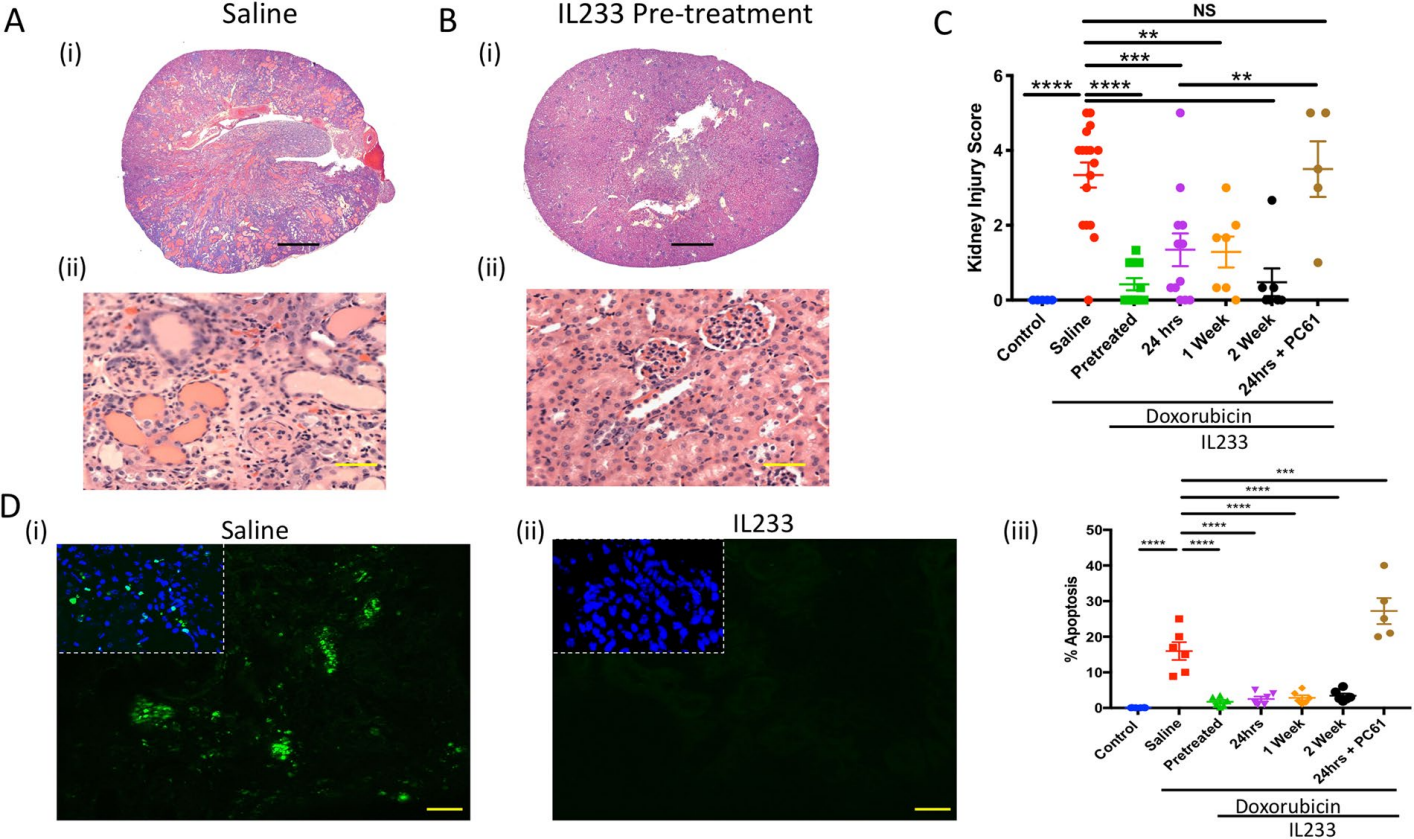
Renal injury was significantly lower upon IL233 treatment. (**A**) Representative hematoxylin and eosin stained sections (A, iii) Saline, (B, i-ii) IL233 Pre-treatment, Scale bars 300 μm (A-i and B-i), 50 μm (A-ii and B-ii); (**C**) Kidney injury score; (**D**) Representative images for TUNEL assay to analyze apoptosis, (i) Saline, (ii) pre-treatment, (iii) Enumeration of % apoptotic cells per high-power field. Symbols represent individual mice; mean ± SEM is shown. **p < 0.01; ***p < 0.001; ****p < 0.0001; NS p > 0.05 by one-way ANOVA.

**Figure 5:**
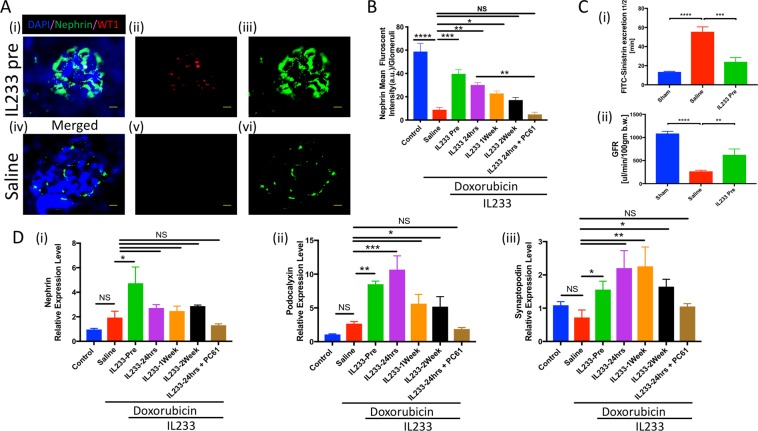
Cytokine Treatment extenuate glomerular injury. (**A**) Immunostaining for Nephrin/WT1 (i, iv) Merged, (ii, v) WT1, (iii, vi) Nephrin; (**B**) Nephrin quantification using ImageJ; (**C**) Measurement of Glomerular filtration (i) Half-life (t_½_), (ii) Glomerular filtration rate; (**D**) Real time PCR analysis of podocyte markers (i) Nephrin, (ii) Podocalyxin, (iii) Synaptopodin. N ≥ 5 for B and D; n ≥ 3 for C; mean ± SEM is shown. *P < 0.05; **P < 0.01; ***P < 0.001; ****p < 0.0001; NS p > 0.05 by one-way ANOVA.

The structural integrity of the kidney was evaluated using H&E stained sections (Fig. 4A,B; Supplementary Fig. 1A–C). Administration of doxorubicin led to both tubular and glomerular damage. The extent of tubular necrosis, cast formation, glomerular hypertrophy, and glomerulosclerosis was  highly pronounced in the saline group as compared to all the IL233-treated groups, including 2-weeks post-doxorubicin group as quantified by blinded Kidney Injury Score (Fig. 4C). When compared to 24 hrs post-treated group, the group injected with PC61 antibody following 24hrs post-treatment resulted in escalation of kidney injury to the levels of saline group. Analysis of cell death (Fig. 4D) using tunnel staining of kidney tissues indicated the presence of significantly higher numbers of TUNEL^+^ cells in the saline-treated as compared to the IL233 pre- and all the post-doxorubicin treated mice. Further, quantitative gene expression analysis and immunostaining data suggest that glomerular structure is well preserved in IL233 treated group. Blocking Treg/ILC2 using PC61 antibody led to the loss of podocytes as indicated by reduced nephrin, synaptopodin and podocalyxin expression (Fig. 5A,B,D). Transcutaneous measurement of GFR using FITC-sinistrin confirms loss of glomerular function in response to doxorubicin in saline control mice, whereas treatment with before doxorubicin maintained GFR with little loss of function (Fig. 5C).

### IL233 treated mice exhibit reduced fibrosis in response to doxorubicin

**Figure 6:**
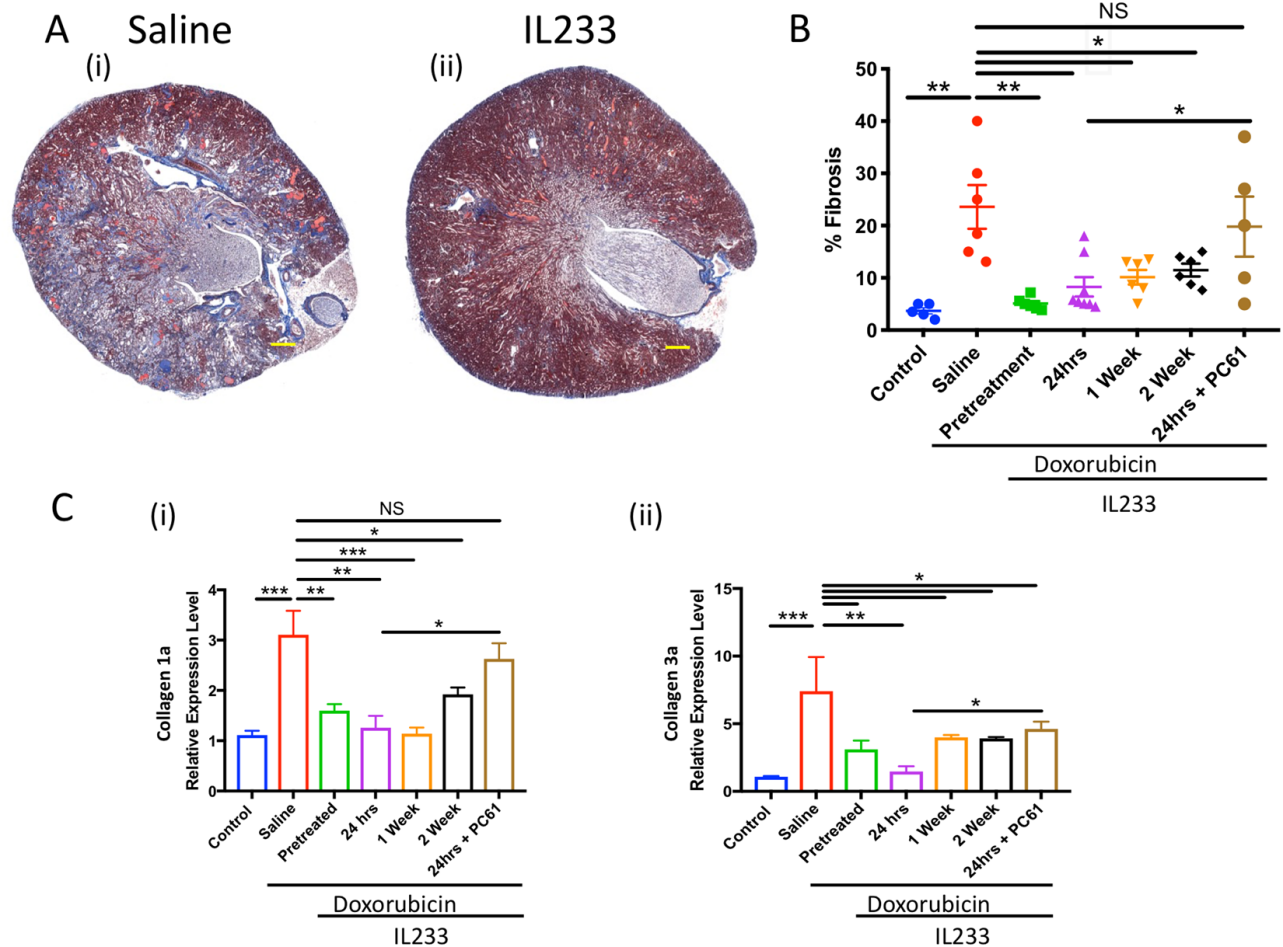
Treatment with IL233 attenuates fibrosis. (**A**) Representative image of Masson’s-Trichrome staining, (i) Saline, (ii) IL233 pre-treatment, Scale bars 300 μm (i–ii); (**B**) Quantification of fibrosis using ImageJ; (**C**) Real time PCR analysis of fibrosis markers (i) Collagen 1a, (ii) Collagen 3a, day 28; Symbols represent individual mice; n ≥ 5 for (**C**); mean ± SEM is shown. *P < 0.05; **P < 0.01; ***P < 0.001; ****p < 0.0001; NS p > 0.05 by one-way ANOVA.

Masson’s-Trichrome staining (Fig. 6A) was performed to examine the levels of fibrosis. Quantification of trichrome staining using ImageJ software (Fig. 6B) showed that treatment with all regimen of IL233 strongly and significantly attenuated fibrosis as compared to saline. Further analysis using qPCR demonstrated that markers of fibrosis, collagen 1 (Fig. 6C-i), collagen 3 (Fig. 6C-ii), alpha2 smooth muscle actin and vimentin (Supplementary Fig. 2A) were upregulated in the saline group as compared to untreated controls. All the IL233 treatment strategies significantly attenuated fibrosis in the doxorubicin injected mice. Importantly, the expression of these genes even in the 2-weeks post-doxorubicin treatment was not different from the naïve control mice indicating complete regeneration and attenuation of fibrosis.

### Increased expression of progenitor and transporter cell genes in IL233 treated mice

**Figure 7:**
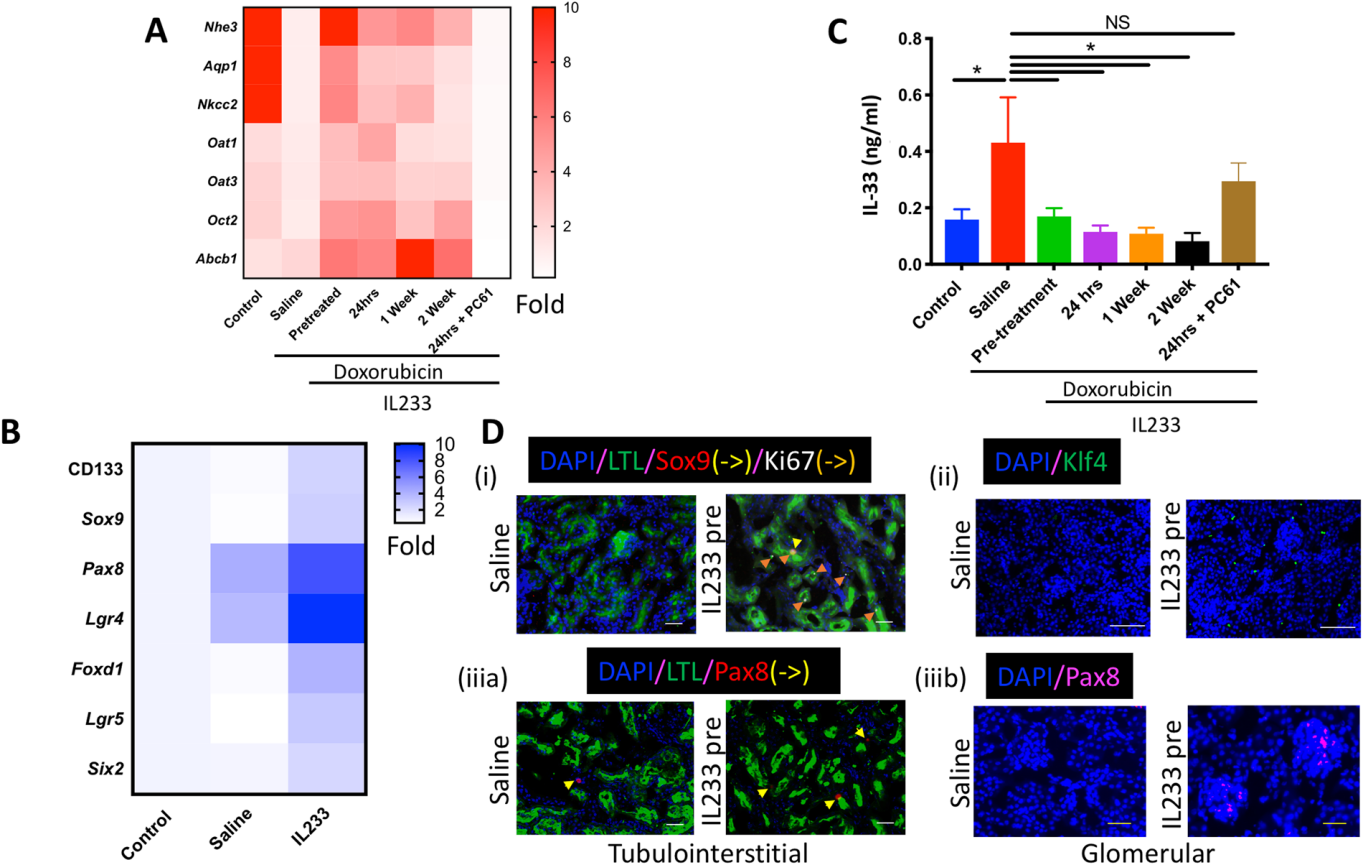
IL233 treatment results in higher transporter and progenitor gene expression in kidneys. Real time PCR analysis of (**A**) renal transporter gene; (**B**) kidney progenitor genes (also see Supplementary Fig. 2); (**C**) levels of alarmin cytokine – IL-33. (**D**) Immunostaining of Saline/IL233-Pre (i) LTL/Sox9/ki67, (ii) Klf4, (iii) LTL/Pax8; Scale: 100 μm. N ≥ 6 except n = 4 for control in panel C, day 28; mean ± SEM is shown. *p < 0.05; NS p > 0.05 by one-way ANOVA.

Compared to naïve controls, mice treated with doxorubicin not only had increased renal injury (Figs. 1 and 5) and fibrosis (Fig. 6), but they also had lower expression of the genes for nephron-associated transporter proteins sodium–hydrogen exchanger 3 (*Nhe3*), Aquaporin 1 (*Aqp1*) and Na-K-Cl cotransporter (*Nkcc2*), indicating loss of multiple nephron segments (Fig. 7A). In comparison, mice treated with IL233 had preserved renal function (Figs 1 and 5C), intact renal architecture (Figs 4, 5), lower fibrosis (Fig. 6), and importantly, increased expression of genes for transporter proteins along the entire nephron (Fig. 7A and Supplementary Fig. 2) as compared to the saline-treated mice, thus, indicating that functional renal integrity was preserved upon IL233 treatment, which was ameliorated by the Treg-inhibiting PC61 antibody. We also analyzed the expression of other relevant renal transporters including organic anion transporter 1 (*Oat1*), organic anion transporter 3 (*Oat3*), organic cation transporter 2 (*Oct2*) and multidrug ABC transporter *Abcb1* (p-gp), which have been known to influence uptake of antibiotics and NSAIDS by the proximal tubular cells^21^. Among them *Abcb1* has been shown to influence uptake of doxorubicin^22^. Treatment with doxorubicin induced a reduction in the expression of *Oat1*, *Oat3* and *Oct2*, but did not influence the expression of *Abcb1*. Treatment with IL233 resulted in an increase in the expression of *Oat1*, *Oat3*, *Oct2* and *Abcb1* in the kidneys of doxorubicin treated mice (Fig. 7A and Supplementary Fig. 2). Interestingly, the expression of several kidney progenitor marker genes, *Cd133*, *Sox9*, *Pax8*, *Lgr4*, *Foxd1*, *Lgr5* and *Six2* (Fig. 7B) was also higher in the IL233 administered group compared to the saline. Expression of *Pax8* and *Lgr4*, which are associated with ureteric bud development, was upregulated in the mice treated with doxorubicin and saline indicating initiation of a reparative program upon renal injury. Treatment with IL233 further enhanced the expression of these genes. Among the treated groups (Supplementary Fig. 2) expression of progenitor cell markers was highly upregulated in the pretreated group compared to post-treatment. The gene expression data corroborated with immunostaining data indicating higher expression of *Sox9*, *Pax8*, *Klf4*, *Ki67 and WT1* positive cells (Figs 5A and 7D). Thus, connoting that pre-treatment with the IL233 hybrid cytokine resulted in the formation of a conducive niche for regeneration of renal tissue after injury. IL-33 is an “alarmin cytokine” that belongs to the IL-1 family. It is upregulated during the tissue injury thus directly indicating the levels of damage to the tissue^23^ and was found to get upregulated in cisplatin-induced renal injury^24^. We observed that IL-33 level was increased in kidneys upon doxorubicin-induced injury. Further, treatment with IL233 resulted in lower IL-33 levels indicating reduced renal injury.

## Discussion

Drug-induced nephrotoxicity is a major concern in the administration of chemotherapeutic agents. Anticancer therapy generally is not directed, hence it affects multiple organs including the kidneys, leading to acute kidney injury (AKI), renal dysfunction, chronic kidney disease (CKD) and end-stage renal disease (ESRD), which is a huge medical burden^25,26^. Development of novel regenerative techniques might prevent the progression of AKI to CKD. Doxorubicin is member of the anthracycline class of antitumor drugs with a wide spectrum of activity in human cancers. Administration of doxorubicin in mice resulted in increased renal inflammation, glomerular hypertrophy, glomerulosclerosis, tubular necrosis, cast formation and deterioration of renal function. The exact mechanism of doxorubicin-induced nephrotoxicity is however unknown^18^. Previous studies have demonstrated that regulatory T-cells (Treg) play a vital role in suppressing innate and adaptive immune response-mediated inflammation and facilitating recovery following renal injury^3,27^.

IL-2 plays an important role in the homeostasis of Tregs^8,28^. We found that IL-2 controls the expression of the IL-33 receptor ST2^29^. IL-33 has been demonstrated to activate Th2 cells and ILC2^30^. We observed synergistic actions of IL-2 and IL-33 to enhance Tregs and generated a hybrid cytokine (IL233), which contains IL-2 and IL-33 activities in a single molecule and was more efficient than IL-2 and IL-33 administered either alone or as a mixture, to increase Tregs and protect mice from IRI^14^. In this study, we evaluated the protective efficacy of IL233 administered either before or after doxorubicin-induced renal injury. Treatment with IL233 either before or after doxorubicin treatment potently rescued renal function measured by plasma creatinine and BUN levels^31^. IL233 treatment also inhibited renal injury as analyzed by improved histology and reduced expression of markers of renal injury, *Kim1* and *Ngal*^32^ compared to saline control.

Adoptive transfer of mice with wild-type Tregs injected before doxorubicin injection protected from renal dysfunction and damage^3^. Further, CD25^+^ Tregs from human CD39-transgenic mice offered greater protection from doxorubicin-induced nephropathy than wild-type CD25^+^ Tregs^33^. Interestingly, injection of T-cells transduced retrovirally with *Foxp3* gene one week after doxorubicin injection also protected from renal dysfunction and injury^34^. Recently, Tregs have been shown to promote regeneration in injury models^35^. Tregs may contribute to regeneration by exerting their influence on local parenchymal cells or progenitor cells in addition to regulating the activities of macrophages^36^. Blocking of Tregs using PC61 antibody resulted in reduction in number of circulating Tregs and exacerbated kidney injury.

The ILC2 localize near epithelium and react to IL-33 alarmin signals released by damaged or dying epithelial cells. ILC2 has been shown to promote regeneration through secretion of amphiregulin, which binds to the epidermal-growth-factor receptor (EGFR), in turn resulting in cellular proliferation and differentiation^10^. ILC2 were shown to promote repair in lung injury^37^ as well as kidney injury models^38^. Recently, Riedel *et al*. demonstrated that treatment of mice with IL-33 for 4-consecutive days starting 5-days after doxorubicin protected from glomerulosclerosis, as evaluated on day14 post-doxorubicin injection, when the kidneys of control mice were injured and dysfunctional^39^. In our study, IL233 therapy starting at this time point (2 weeks after doxorubicin injection), completely protected mice from kidney damage and dysfunction when analyzed further 2 weeks later, suggesting that increases in Tregs and ILC2 with IL233 therapy, very late in the injury has the potential to induce recovery from renal injury and regain function. Thus, implying that increase in Treg/ILC2 could have contributed towards rescuing the function of the kidney. The mechanism by which Tregs and ILC2 collude together to promote regeneration is not completely understood, though, both cell-types have been shown to a produce amphiregulin, and contributes towards regeneration of epithelial cells^12,40^.

Within hours of injury, leukocytes such as neutrophils and macrophages, followed by CD4^+^ and CD8^+^ T cells infiltrate the target organ inducing inflammation, which modulates injury and repair^41,42^. These cells secrete an array of pro-inflammatory or anti-inflammatory cytokines, which can either aid or impede regeneration depending on the immunological milieu surrounding the damaged microenvironment. Both in chronic and acute injury there is an enhanced presence of Tregs, constituting about 40–50% of CD4^+^ T cells which accumulate in the injured region and contribute towards regeneration^35,43^. In our study, it is possible that acute renal injury persists as late as 2 weeks post-doxorubicin administration and the enhancement of Tregs with IL233 at these time points may still be preventing the continuation of acute injury and its progression to chronic injury. Damage associated molecular pattern (DAMP) and chemokines released from sterile kidney injury mostly drive the inflammation process with the earliest influx of cells of the innate immune system including predominantly neutrophils, monocytes/macrophages and natural killer (NK) T cells^44^, later-on followed by T-cells^45^. These cells have been shown to secrete IFNγ and TNFα that contribute to renal injury in a positive feedback loop^46^. Treatment with IL233 hybrid cytokine resulted in significant attenuation of the production of the pro-inflammatory cytokines IFNγ and TNFα and increase in production of anti-inflammatory cytokine IL-10 by many cell types including Tregs, and has been shown to be important in restricting inflammation associated with renal injury^2,47^. This effect could promote repair pathways thus indirectly contributing towards regeneration, preventing the structural damage and protecting the function of the kidney. Riedel *et al*. showed that IL-33 treatment increased IL-5 and IL-13 producing ILC2 in the kidneys, which induced recruitment of eosinophils and skewing towards M2 macrophages, which prevented renal dysfunction^39^. In our study we observed that levels of Th2 cytokines, IL-5 and IL-13 were also significantly upregulated in multiple post-treatment groups (Supplementary Fig. 6). Another recent study demonstrated a role for IL-33 and ILC2 in protecting naive as well as humanized mice from renal IRI^48^. However, in that study administration of anti-CD25 PC61 antibody did not alter the IL-33 mediated protective responses. Although, we are using a different model system and the IL233 hybrid cytokine (bearing both IL-2 and IL-33 activities), PC61 treatment in our study abrogated the renal protection. PC61 treatment not only reduced the levels of Tregs, but also reduced ILC2 levels in the spleens, likely owing to the expression of CD25 on ILC2^49^. Interestingly, PC61 treatment did not alter the renal levels of Tregs and ILC2 in the IL233 treated mice, despite the loss of renal protection indicating that PC61 may deplete Tregs and ILC2 in the lymphoid organs and inhibit their function in the tissues^50^. Another possibility is a rapid repopulation of either Foxp3^+^ Tregs and ILC2 in the injured organs from primary lymphoid organs, once the effects of PC61-blockade disappears or if this subpopulation of Foxp3^+^ Tregs and ILC2 did not express CD25, when PC61 was injected^51^. In our future studies, we will investigate the specific downstream pathways affected by PC61 in IL233 treatment. We observed that doxorubicin increased the levels of IL-33 in kidneys, which could be an innate signal for recruitment of Tregs and ILC2 to the injured kidneys. Treatment with IL233 in pharmacological doses would strengthen this recruitment and offer protection.

As a consequence of doxorubicin-induced kidney injury, the normal architecture is disrupted, including changes in the tubular brush border and glomerular morphology^52^. Further, in maladaptive renal repair, as observed in saline-treated controls, there is activation of myofibroblasts, resulting in severe fibrosis. In mice treated with IL233, the majority of the kidneys appeared indistinguishable from uninjured kidneys. Our data suggest that treatment with IL233 attenuated both inflammation and fibrosis while restoring normal renal function. Sodium-hydrogen antiporter 3 (NHE3) that is involved in maintaining the sodium balance, is found on the apical side of proximal tubule epithelium and thick ascending limb^53,54^. Aquaporin 1 (AQP1) is a major water transport channel protein^55^. It is found in basolateral and apical side of proximal tubule, descending limb of loop of Henle and descending portion of vasa recta. NKCC2 is a membrane transport protein that transport sodium, potassium and chloride into across the cell membrane^56^. We also observed that in addition to the above transporter’s renal injury, doxorubicin also decreases the expression of organic anion and action transporters (*Oat1*, *Oat3*, *Oct2* and *Abcb1*) in the kidneys and treatment with IL233 either before or after the injury restored their expression. These have been implicated in altering the transport/clearance of several drugs^21,22^. It is possible the pretreatment with IL233, which restored the structure and function of renal tubules also preserve the expression of these transporters enabling rapid clearance of doxorubicin. However, treatment with IL233 as late as 1 week or 2 weeks post doxorubicin showed high expression of these transporters indicate restoration of renal tubular function (Fig. 7A). At these late time points the majority of doxorubicin must have been cleared, because the retention half-lives in heart, lung, kidney and small intestine were reported between 4.5 to 6.5 hours^57^ suggesting that modulation of the expression of organic transporters in IL233 treated mice may serve as an indicator of renal functional integrity, most likely independent of doxorubicin clearance. Thus, our data imply that Tregs and ILC2 play a vital role in protecting the structure and function of the kidney, along with an increased transporter gene expression (Fig. 7A) as compared to the saline-controls besides preventing the maladaptive repair and fibrosis.

Following limited kidney injury, rapid and spontaneous repair and regeneration of renal tissue occurs^58^. Two hypotheses explain the mechanism of regeneration. First, cellular plasticity, where the adult cells have the ability to dedifferentiate into progenitor cells with in turn differentiate into another cell type. Second, progenitor cells, where the adult kidney tissue maintain some undifferentiated multipotent progenitor cells which are activated upon injury giving rise to specialized cell types. For this study, we have narrowed down the panel of progenitor markers to *Sox9*, *Pax8*, *Lgr4*, *Lgr5*, *Foxd1*, *Six2*, *WT1*, *CD 133* and *CD90* to investigate the potential role of progenitor cells in regeneration. Sex-Determining-Region Y-Box 9 protein (*Sox9*) is a transcription factor expressed in a set of progenitor cell population that has been recently reported to be involved in renal repair process^59^. Cap mesenchymal (CM) cells are a class of stem cells that has self-renewal capability and gives rise to different structures of the nephron^60–62^. CM consists of unique combination of transcription factors such as *Pax2*, *Pax8*, *Six2* and *WT1*. Although these transcription factors are down regulated in adult mammalian kidneys, they have been shown to be upregulated in injured kidney during the onset of reparative program. Recently, *Lgr4/Lgr5* expressing progenitor cell populations was shown to be involved in renal development and regenerative program^63,64^. Foxd1 expressing stromal cell progenitors are derived from metanephric mesenchymal cells that gives rise to interstitial and mesangial cells^65^. Additional some of cells stromal as well as epithelial cells expressing miscellaneous markers such as CD133, CD25 and CD90 has been reported to contribute towards the repair^65^. In this study, we observed that following renal injury expression of progenitor cell related genes *Pax8* and *Lgr4* were slightly upregulated (Fig. 7B), indicating the onset of a reparative or regenerative program. However, in the IL233 treated group the expression of not only *Pax8* and *Lgr4* was further upregulated, but several other renal progenitor genes including *CD133*, *Sox9*, *Lgr5*, *Foxd1*, and *Six2* was significantly elevated, which could have contributed towards enhanced regeneration. The data suggest that either Tregs, ILC2 or both cell types are invoked by IL233 and may promote the recruitment of progenitor cells either directly or through inducing an anti-inflammatory milieu, which may be conducive for progenitor cells to promote the regeneration mechanisms.

In conclusion, early and precise intervention is mandatory to control progression of nephropathy. The current data shows that fusion cytokine IL233 increases the levels of Treg and ILC2 in lymphoid as well as the injured organs to restrict inflammation to prevent further damage to the affected organ and also initiate a reparative program for regeneration of the injured kidney. Importantly, intervention as late as 2 weeks post-doxorubicin administration led to restoring the function of the kidney, prevented structural damage, and reduced the inflammation. Treatment with IL233 cytokine may result in the formation of a protective niche that might rescue kidney from various external insults. This is the first evidence of an immunomodulatory cytokine that was employed therapeutically as late as 2-weeks post-induction of injury resulting not only in attenuation of inflammation, but initiation of a regenerative program marked with high expression of progenitor cell markers. Thus, IL233 hybrid cytokine may be a potential therapeutic agent to rescue the kidney from injury including nephrotoxic drugs and other renal insults.

## Methods

### Animal model

All experimental procedures with animals were carried out in accordance with NIH Guide for the Care and Use of Laboratory Animals and approved by University of Virginia Animal Care and Use Committee. Male BALB/cJ mice (8–12 weeks old) were purchased from Jackson Laboratory (Bar Harbor, USA). Nephrotoxicity was induced by intravenous (i.v.) injection of doxorubicin hydrochloride (Sigma Chemical Company) at a dose of 10 mg/kg body weight in 0.9% saline. The mice were injected with IL233 at 3.3pmol/g/d intraperitoneally (i.p.) for 5-consecutive days starting either 8 days before, 24 hours after, 1 week after or 2 weeks after doxorubicin administration. The IL233 hybrid cytokine was produced as described previously^14^. Mice were euthanized at day 28 after doxorubicin treatment for further analysis. A subset of mice in the 24-hour IL233 treatment group were administered with anti-mouse CD25 monoclonal antibody (PC61) three times with 100μg intraperitoneally every other day following cytokine injection.

### Kidney function analysis

The mice were anesthetized with Avertin (0.5 mg/g, i.p.) and blood was collected retro-orbitally. Plasma separated from the blood was used for plasma creatinine (PCr) and blood urea nitrogen (BUN) analysis. Enzymatic method (Diazyme Laboratories) was used for measurement of PCr levels with a modification to the manufacturer’s instructions that the volume of sample was doubled. BUN levels were measured using a colorimetric assay kit according to manufacturer’s protocol (Arbor Assays). Glomerular filtration rate (GFR) was determined in conscious mice using transcutaneous measurement of fluorescein isothiocyanate (FITC) elimination kinetics. The procedure was carried out as previously described by Perez *et al*.^66^.

### Histology and assessment of kidney injury and fibrosis

Transverse sections of kidneys (5μM) fixed in 10% buffered formalin were stained with hematoxylin and eosin (H&E) and examined using light microscopy for evaluation of kidney injury. Blinded kidney injury score was used to quantify the level of kidney injury by examining three random areas of each kidney section. The score was classified as no injury (0), mild with damage in <20% of the section (1), moderate with damage in 20–40% of the section (2), high 40–60% damage in the section (3), extensive with 60–80% damage in the section (4) and severe with >80% damage in the section (5) based on the assessment of levels of tubular necrosis, cast formation, glomerular hypertrophy and glomerulosclerosis. The level of fibrosis in the kidney was evaluated histologically using Masson’s Trichrome staining. Fibrotic areas from the sections were quantified using ImageJ software.

### Immunofluorescence

Kidneys were fixed with 1% PLP for 24 hours and 15% sucrose for 3 hours at 4 °C, embedded and frozen in Optimal Cutting Temperature (OCT) compound (Ted Pella, Inc.). Tissue were sectioned 5 μm thick, permeabilized with 0.2% Triton X-100 and blocked using 1% fetal bovine serum. If using unconjugated antibody, samples were first incubated with primary antibody for overnight at 4 °C and subsequently incubated with fluorescent dye conjugated secondary antibody for 4hrs at room temperature. All the samples were mounted using ProLong Diamond Antifade (Thermo Fischer Scientific) containing 4′,6-diamidino-2-phenylindole (DAPI) counterstain. Images were acquired using the Carl Zeiss Axiovert 200 microscopy system with Apotome imaging and Axiovision software (Car Zeiss Microscopy, LLC).

### Gene expression analysis

Total RNA was isolated using Trizol (Invitrogen, CA, USA) and iScript cDNA synthesis kit (BioRad) was used to prepare cDNA. Real time PCR analysis of samples was carried out using iQ SYBRGreen Supermix (BioRad) or Taqman Assay (Thermo Fischer Scientific) in CFX Real-Time PCR Detection System (BioRad). The housekeeping gene *GAPDH* was used to normalize the gene expression levels. Primer sequences used to quantify the endogenous gene expression levels are listed in the Supplementary Table 1.

### Flow Cytometry

Flow cytometry was performed as described previously^2,14^. Antibodies used for analysis are listed in Supplementary Table 2. Data were acquired on a FACScan cytometer (BD Biosciences) with a 5-color upgrade (Cytek Development, Inc.) and analyzed with FlowJo software using the gating strategies shown in Supplementary Figs 3, 4 and 5 (TreeStar, Inc.).

### Apoptosis

TUNEL assay, *In Situ* Cell Death Detection Kit, and Fluorescein (ROCHE) were used to detect the levels of apoptosis in kidney tissues. The procedure was carried out as per the manufacturer’s instructions on 5 μM snap-frozen tissue sections. The images were acquired using the Carl Zeiss Axiovert 200 microscopy system with Apotome imaging and Axiovision software (Car Zeiss Microscopy, LLC) and the proportion of apoptotic cells identified as green fluorescence intensity within the DAPI fluorescence intensity were quantified in the entire kidney section with the help of ImageJ software.

### Cytokine Assay

IL-33 cytokine levels were determined from kidney tissue lysate using commercial ELISA kit (Thermo Fisher Scientific) as per manufacturer’s instructions. Cytokine concentrations in the kidney lysate were normalized to total protein content measured by BCA Assay (Thermo Fisher Scientific).

### Statistics

Data are the summary of five independent experiments. Statistical analysis of comparison between multiple groups was carried out using one-way ANOVA followed by Dunnett’s P test or Tukey test using GraphPad Prism 7 software. Results are expressed as the mean ± standard error. Results with p < 0.05 were considered significant. P < 0.05 (*), p < 0.01(**), p < 0.001(***), p < 0.0001(****), NS p > 0.05. Additional information on number and reproducibility of the data are in the Supplementary Statistical Analysis and Reproducibility section of the manuscript.

## Supplementary information

Supplementary information

## Data Availability

All relevant data are detailed in the manuscript. Additional information on materials, data and associated protocols will be available to readers upon requested.
