# Supplementary material for: A Novel Hybrid Cytokine IL233 Mediates regeneration following Doxorubicin-Induced Nephrotoxic Injury
Source: Sci Rep. 2019 Mar 1;9:3215. doi: 10.1038/s41598-019-39886-9 (PMC6397151; doi:10.1038/s41598-019-39886-9)

# A NOVEL HYBRID CYTOKINE IL233 MEDIATES REGENERATION FOLLOWING DOXORUBICIN-INDUCED NEPHROTOXIC INJURY

Vikram Sabapathy, Nardos Tesfaye Cheru, Rebecca Corey, Saleh Mohammad, and Rahul Sharma

## Supplementary Information

**Supplementary Table1: QPCR Primers**

| Primers | Sequence 5'-3' or Taqman Catalog no. |
|---|---|
| Kim1 Fwd | ACATATCGTGGAATCACAACGAC |
| Kim1 Rev | ACTGCTCTTCTGATAGGTGACA |
| Ngal Fwd | TGGCCCTGAGTGTCATGTG |
| Ngal Rev | CTCTTGTAGCTCATAGATGGTGC |
| Col1a1 Fwd | GCTCTTTTTAGATACTGTGGTGAGGAA |
| Col1a1 Rev | GTTTCCACGTCTCACCATTG |
| Col3a1 Fwd | ACAGCTGGTGAACCTGGAAG |
| Col3a1 Rev | ACCAGGAGATCCATCTCGAC |
| Vimentin Fwd | GATCGATGTGGACGTTTCCAA |
| Vimentin Rev | ATACTGCTGGCGCACATCAC |
| Acta2 Fwd | CTGACAGAGGCACCACTGAA |
| Acta2 Rev | AGAGGCATAGAGGGACAGCA |
| Nhe3 Fwd | TGCCTTGGTGGTACTTCTGG |
| Nhe3 Rev | TCGCTCCTCTTCACCTTCAG |
| Aqp1 Fwd | CCGAGACTTAGGTGGCTCAG |
| Aqp1 Rev | TGATACCGCAGCCAGTGTAG |
| Nkcc2 Fwd | GGCTTGATCTTTGCTTTTGC |
| Nkcc2 Rev | CCATCATTGAATCGCTCTCC |
| CD133 Fwd | CCCGGAGGAGAGCTGGACCG |
| CD133 Rev | AGGGCAATCTTCAGAGCCAGACT |
| Sox9 Fwd | GTGCAAGCTGGCAAAGTTGA |
| Sox9 Rev | TGCTCAGTTCACCGATGTCC |
| Pax8 Fwd | CAGAAGGCGTTTGTGACAATGA |
| Pax8 Rev | TGCACTTTGGTCCGGATGAT |
| Lgr4 Fwd | CCGCTGCCTGCTTGCCTGAA |
| Lgr4 Rev | TCCTGGTGACACGCCGCTTC |
| Foxd1 Fwd | CCCCTCCTGGACTAACCGGGC |
| Foxd1 Rev | CGAGGTGTTTGCGCTCCCCG |
| Lgr5 Fwd | CACAGCCACTGCGGCGACTT |
| Lgr5 Rev | CAATGGGCGTCTGCCGGGTC |
| Six2 Fwd | GGACGGATCGTTGTGACTCAGGA |
| Six2 Rev | CGCTGTTCTCCCTTTCCTGGC |
| Cd90 Fwd | AGTCTCGGGCGCGAATCCCA |
| Cd90 Rev | TGCCGCCACACTTGACCAGC |
| Nephrin Taqman Probe | Mm01176615_g1 |
| Synaptopodin Fwd | CCTGCCCGTAACTTCCGTG |
| Synaptopodin Rev | GAGCGGCGGTAGGGAAAA |
| Podocalyxin Fwd | TACTGTCGCCTGCATCTCAC |
| Podocalyxin Rev | GTGTCCAAAGCCATCCAGTT |
| Oat1 | Mm00456258_m1 |
| Oat3 | Mm00459534_m1 |
| Oct2 | Mm00457295_m1 |
| Abcb1b | Mm00440736_m1 |

**Supplementary Table2: Antibodies**

| Antigen | Conjugate | Catalog No. | Company |
|---|---|---|---|
| Foxp3 | Alexa 488 | 53-5773-82 | ebioscience |
| ST2 | PE | 145304 | BioLegend |
| CD4 | PercpCy5.5 | 100434 | BioLegend |
| CD25 | APC | 102012 | BioLegend |
| TCRb | APCeflour780 | 47-5961-82 | ebioscience |
| CD90 | FITC | 553013 | BD Biosciences |
| NK1.1 | PercpCy5.5 | 108728 | BioLegend |
| B220 | APC/Fire 750 | 103260 | BioLegend |
| CD3 | APC | 551163 | BD Biosciences |
| CD45 | APC/cy7 | 103115 | BioLegend |
| CD4 | APC-eflour780 | 47-0041-82 | ebioscience |
| CD8a | Percp-eflour710 | 46-0081-80 | ebioscience |
| INFg | FITC | 505806 | BioLegend |
| IL4 | PE | 554435 | BioLegend |
| TNFa | FITC | 11-7321-82 | ebioscience |
| IL10 | PE | 17-7101-81 | ebioscience |
| PC61 | Unconjugated | 102014 | BioLegend |
| Sox9 | Unconjugated | SAB4502834 | Sigma |
| Pax8 | Unconjugated | SC-81353 | Santa Cruz |
| Klf4 | Unconjugated | AF3158 | R&D Systems |
| LTL | Fluorescein | FL-1321 | Vector Labs |
| WT1 | Unconjugated | SC-393498 | Santa Cruz |
| Nephrin | Unconjugated | AF3159 | R&D Systems |
| Ki67 | Alexa 647 | 151206 | BioLegend |

**Supplementary Statistical Analysis and Reproducibility**

Male BALB/cJ mice were purchased from the Jackson laboratories (sold at the maximum age of 8 weeks). According to the published literature on this models, we waited until the animals were 10-12 weeks old before doxorubicin administration. The mice were randomly assigned to the different groups. Two independent investigators generated doxorubicin injury model, collected the tissue samples and the data represents compilation of multiple independent experiments. The qPCR, immunohistochemistry, immunofluorescent, ELISA and flow cytometry analysis were also performed by different independent investigators who were single blinded to experimental and outcome assessment.

Figure 1A: Experimental plan to show different treatment groups. The data is compilation of 5 independent experiments.
Figure 1B-C: Control (n=6), Saline (n=19), Pre-treatment (n=15), 24hrs (n=10), 1Week (n=7), 2Week (n=7) and 24 hrs+PC61 (n=4). Data are mean ± SEM; one-way ANOVA.
Figure 1D: Control (n=6), Saline (n=8), Pre-treatment (n=7), 24hrs (n=7), 1Week (n=7), 2Week (n=7) and 24 hrs+PC61 (n=5). Data are mean ± SEM; one-way ANOVA.

Figure 2, A and C: Control (n=5), Saline (n=15), Pre-treatment (n=11), 24hrs (n=10), 1Week (n=7), 2Week (n=7) and 24 hrs+PC61 (n=4). Data are mean ± SEM; one-way ANOVA.
Figure 2, B and D: Control (n=5), Saline (n=10), Pre-treatment (n=7), 24hrs (n=10), 1Week (n=7), 2Week (n=7) and 24 hrs+PC61 (n=4). Data are mean ± SEM; one-way ANOVA

Figure 3 A-D: Control (n=5), Saline (n=15), Pre-treatment (n=11), 24hrs (n=10), 1Week (n=7), 2Week (n=7) and 24 hrs+PC61 (n=4). Data are mean ± SEM; one-way ANOVA.

Figure 4C: Control (n=6), Saline (n=17), Pre-treatment (n=11), 24hrs (n=10), 1Week (n=7), 2Week (n=7) and 24 hrs+PC61 (n=5). Data are mean ± SEM; one-way ANOVA.
Figure 4D (iii): Control (n=), Saline (n=6), Pre-treatment (n=6), 24hrs (n=6), 1Week (n=6), 2Week (n=6) and 24 hrs+PC61 (n=5). Data are mean ± SEM; one-way ANOVA.

Figure 5: B and D (i, ii and iii): Control (n=), Saline (n=6), Pre-treatment (n=6), 24hrs (n=6), 1Week (n=6), 2Week (n=6) and 24 hrs+PC61 (n=5). Data are mean ± SEM; one-way ANOVA.
Figure 5C(i/ii): Control (n=3), Saline (n=3), IL233 Pre-treatment (n=3). Data are mean ± SEM; one-way ANOVA.

Figure 6B: Control (n=5), Saline (n=6), Pre-treatment (n=8), 24hrs (n=6), 1Week (n=7), 2Week (n=6) and 24 hrs+PC61 (n=5). Data are mean ± SEM; one-way ANOVA.
Figure 6C (i/ii): Control (n=5), Saline (n=6), Pre-treatment (n=6), 24hrs (n=6), 1Week (n=6), 2Week (n=6) and 24 hrs+PC61 (n=5). Data are mean ± SEM; one-way ANOVA.

Figure 7A: Control (n=6), Saline (n=6), Pre-treatment (n=6), 24hrs (n=6), 1Week (n=7), 2Week (n=6) and 24 hrs+PC61 (n=5). Data are mean ± SEM; one-way ANOVA.
Figure 7B: Control (n=6), Saline (n=6), Pre-treatment (n=6). Data are mean + SEM; one-way ANOVA.
Figure 7C: Control (n=4), Saline (n=12), Pre-treatment (n=11), 24hrs (n=6), 1Week (n=7), 2Week (n=6) and 24 hrs+PC61 (n=5). Data are mean ± SEM; one-way ANOVA.

Initial set of experiments involved only saline and pre-treated groups. Samples from the initial experiments were analyzed only for plasma creatine and blood urea nitrogen. Once the pre-treated strategy was optimized we included post-treatment group in rest of the experimental set up accounting for the different mouse numbers in different figures. For the real-time PCR experiments data was generated from a compilation of 2 complete set of experiments containing all the groups. No animals were excluded in the analysis of the

parameters unless they died before obtaining the respective tissues or the tissues were not collected for the specific parameters in some of the earlier experiments. In Figure 7C, sufficient amount of serum could not be available from two of the control mice for the IL-33 ELISA as the serum was used-up in other assays as well as flow cytometry and Masson's trichrome staining was not performed on one control samples in one of the repetitions due to loss of the sample during processing.

All the experimental groups were designed so that physiological, biochemical and molecular parameters could be statistically evaluated. We had 3 drop outs in the saline group and 1 in the PC61 treated group as the animals died before day 28. Animals that survived 28 days post doxorubicin administration were used for analysis to maintain uniformity. Although, all the animals in the vehicle (saline) or the PC61 treated group were expected to lose renal function and become sick, it is unclear why these animals died before the rest of the group members during the course of experiments.

**Supplementary Figure 1: Post injury IL233 Treatment.** (A) representative hematoxylin and eosin stained sections (Ai-ii) IL233-24hrs, (Bi-ii) IL233-1Week, (Ci-ii) IL233-2Week, (Di-ii) IL233-24hrs + PC61, Scale bars 300μm (A, B, C, D-i), 50μm (A, B, C, D-ii).

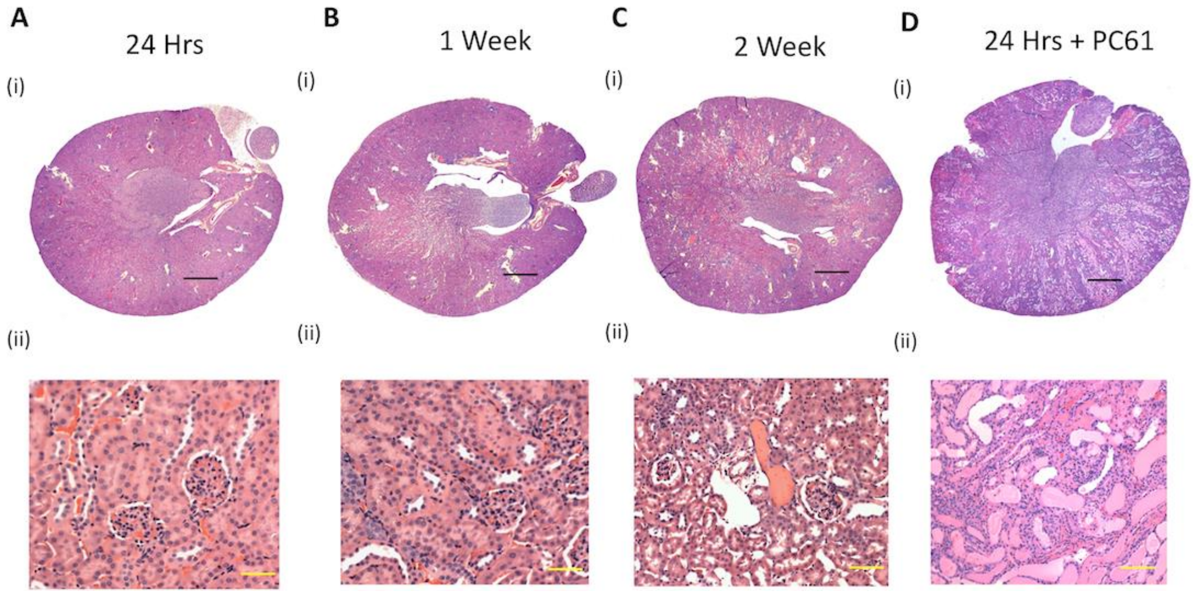

**Supplementary Figure 2:** (A) Real time PCR analysis of fibrosis markers, day 28; (B) Kidney transporters gene expression analysis. (C) Kidney progenitors gene expression. Control (n=6), Saline (n=6), Pre-treatment (n=6), 24hrs (n=6), 1 Week (n=6), 2 Week (n=6) and 24 hrs + PC61 (n=5). Data are mean ± SEM; * p<0.05; ** p<0.01; *** p<0.001; **** p<0.0001; NS p>0.05 by one-way ANOVA.

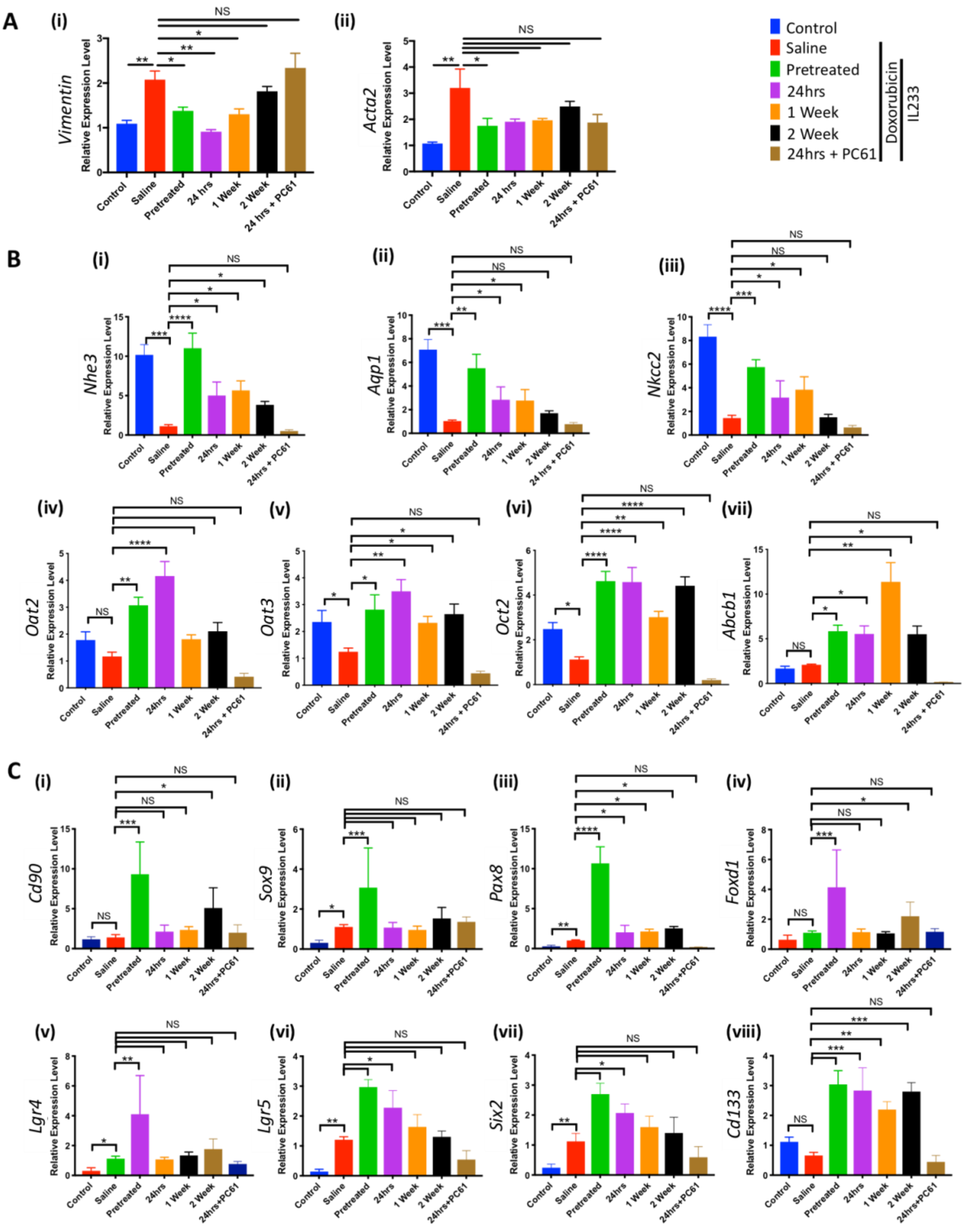

**Supplementary Figure 3**: Treg gating strategy in blood, spleen and kidney

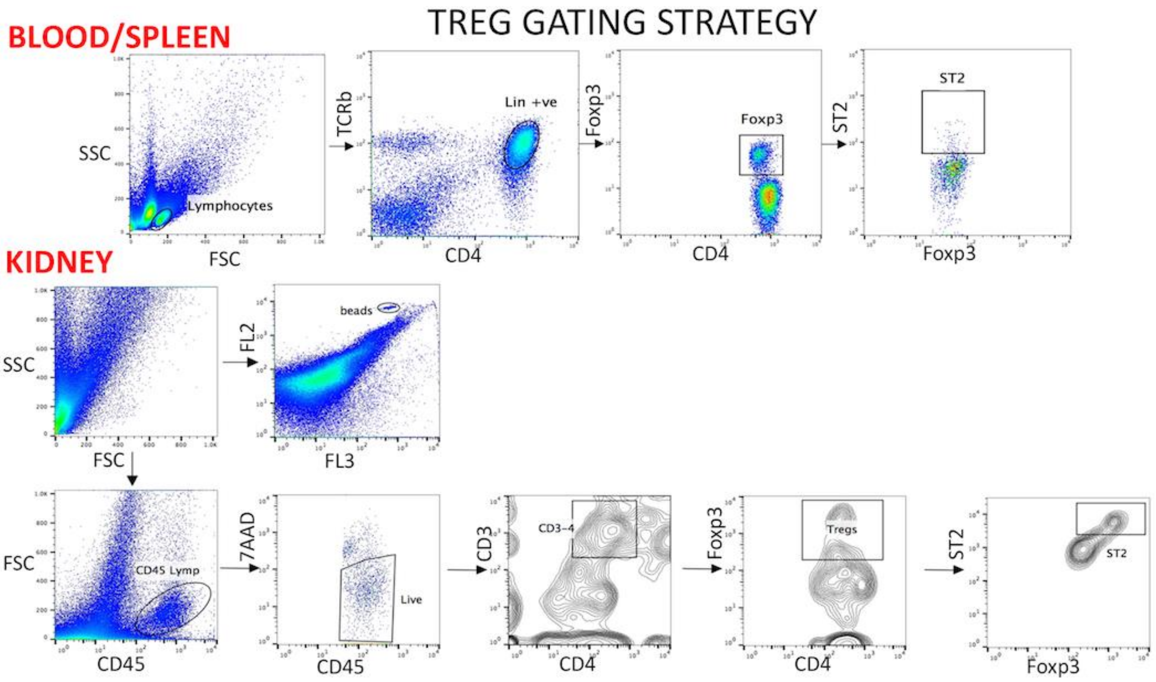


**Supplementary Figure 4**: ILC2 gating strategy in blood, spleen and kidney

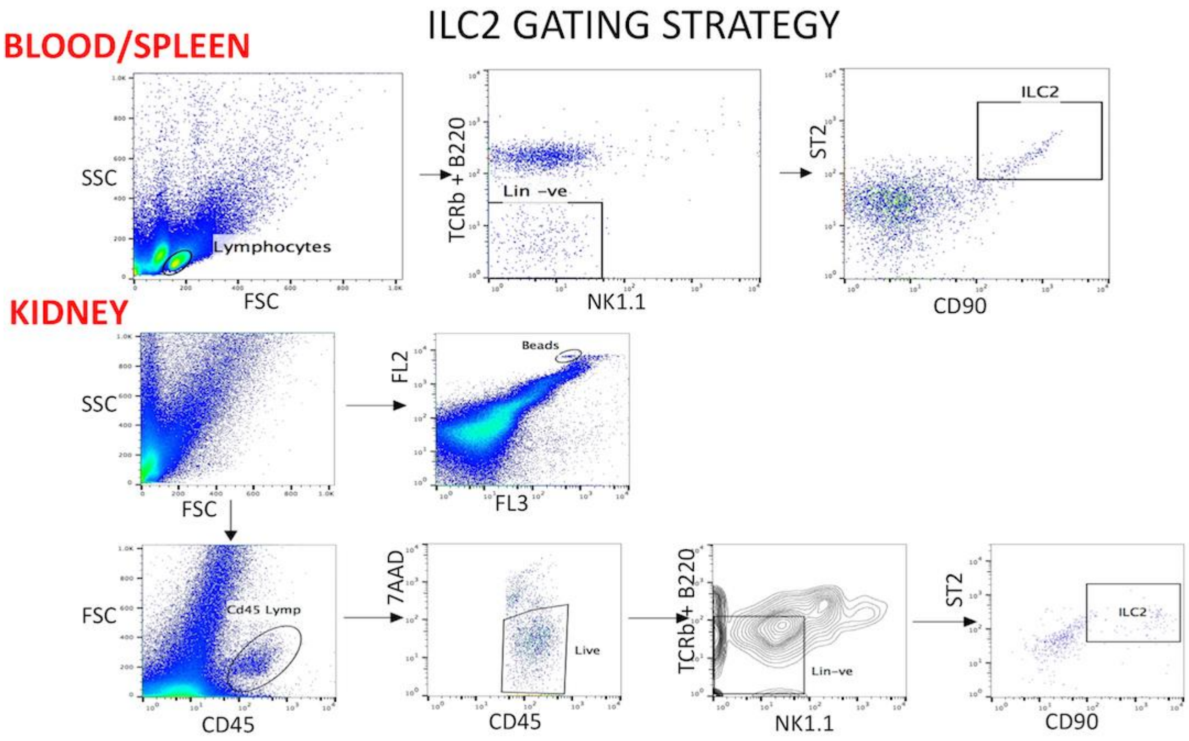

**Supplementary Figure 5**: Cytokine gating strategy

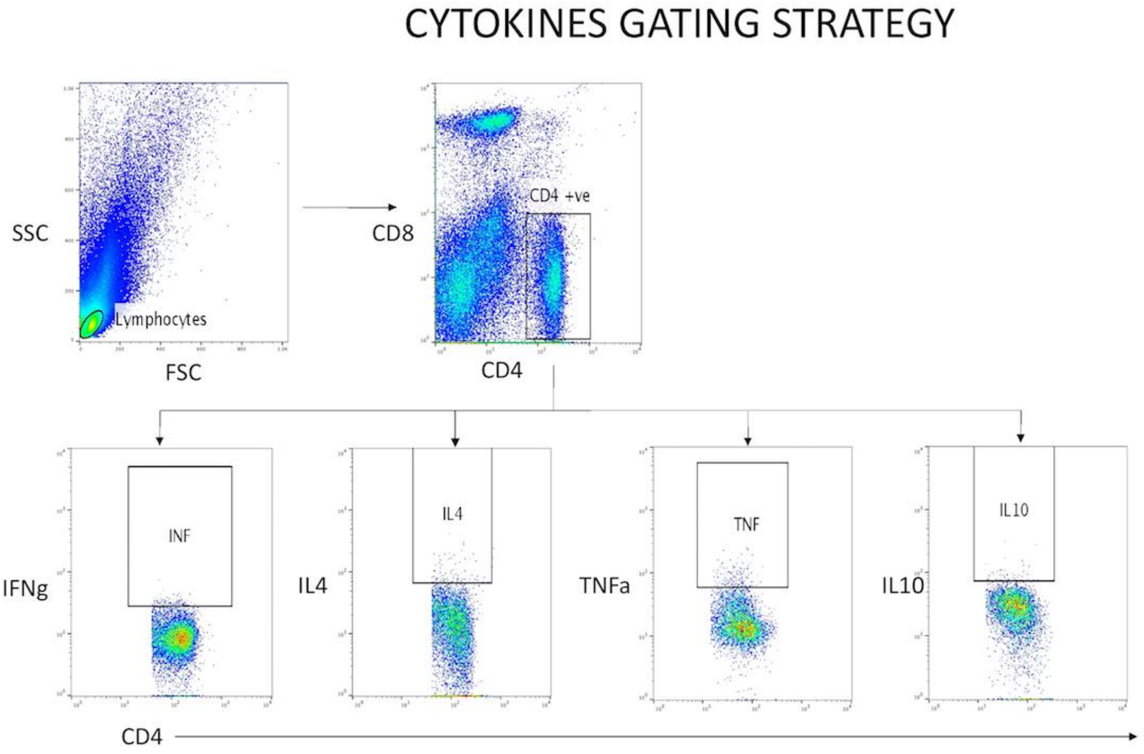


**Supplementary Figure 6**: Flow cytometry analysis in peripheral blood (A) Treg, (B) ILC2; Cytokines (C) IL-5$^+$CD4$^+$, (D) IL-13$^+$CD4$^+$; Flow cytometry analysis of splenic Tregs Day 14 and Day 28 (E). Symbols represent individual mice; mean ± SEM is shown in E (N≥3). * p<0.05; ** p<0.01; **** p<0.0001; NS p>0.05 by one-way ANOVA.

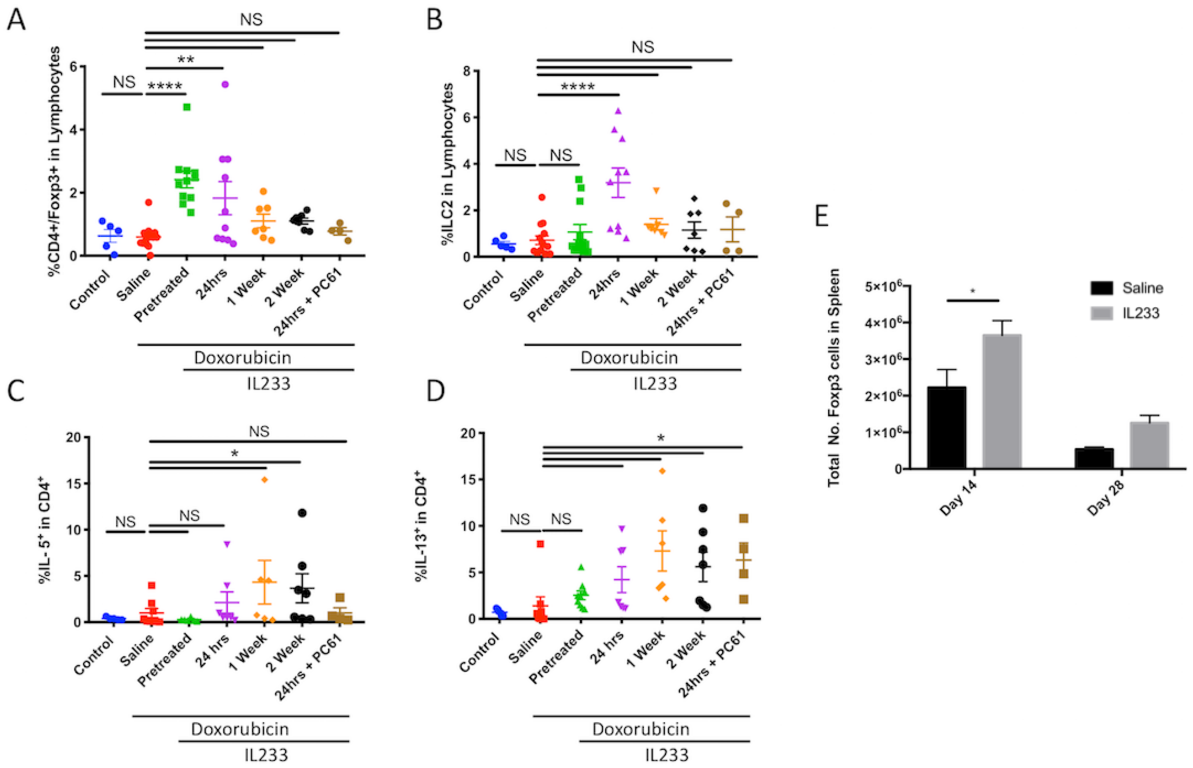

**Supplementary Figure 7**: Time line of Tubular and Tubulo-interstitial pathology in saline treated group.  Scale bars 300μm (A, B, C, D-i), 50μm (A, B, C, D-ii).

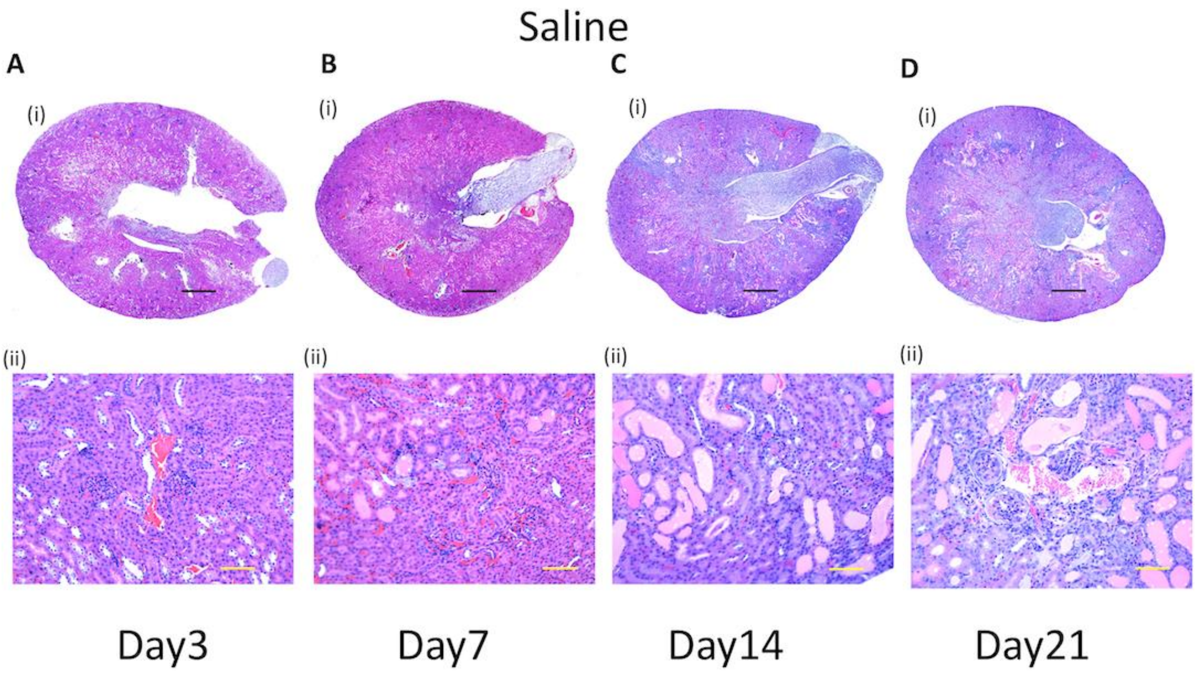


**Supplementary Figure 8**: Time line of Tubular and Tubulo-interstitial pathology in IL233 pretreated group. Scale bars 300μm (A, B, C, D-i), 50μm (A, B, C, D-ii).

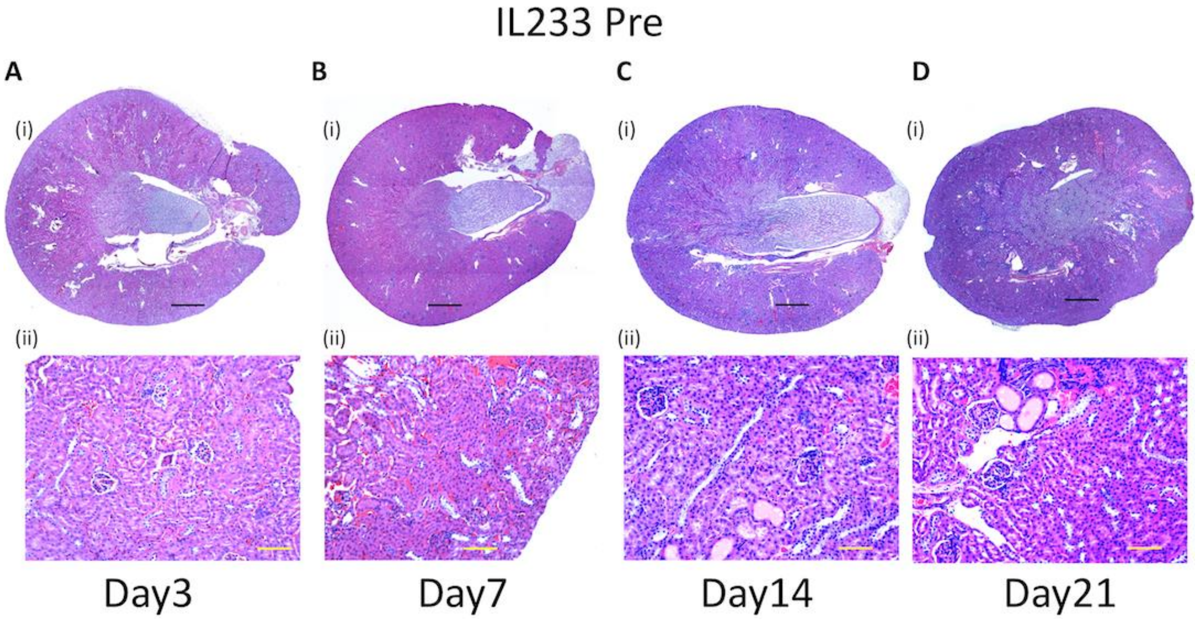

**Supplementary Figure 9**: Time line of Glomerular pathology in saline/IL233 pretreated group. Scale bars 20μm (A, B, C, D- i, ii); (F) Glomerular Injury Score – Quantitated (1-5) based on Glomerular Hypertrophy, Glomerular Sclerosis, Mesangial Expansion, Mesangiolysis and inflammation; (G) Glomerular Perimeter. N=6; mean ± SEM is shown. * p<0.05; *** p<0.001; **** p<0.0001; NS p>0.05 by one-way ANOVA.

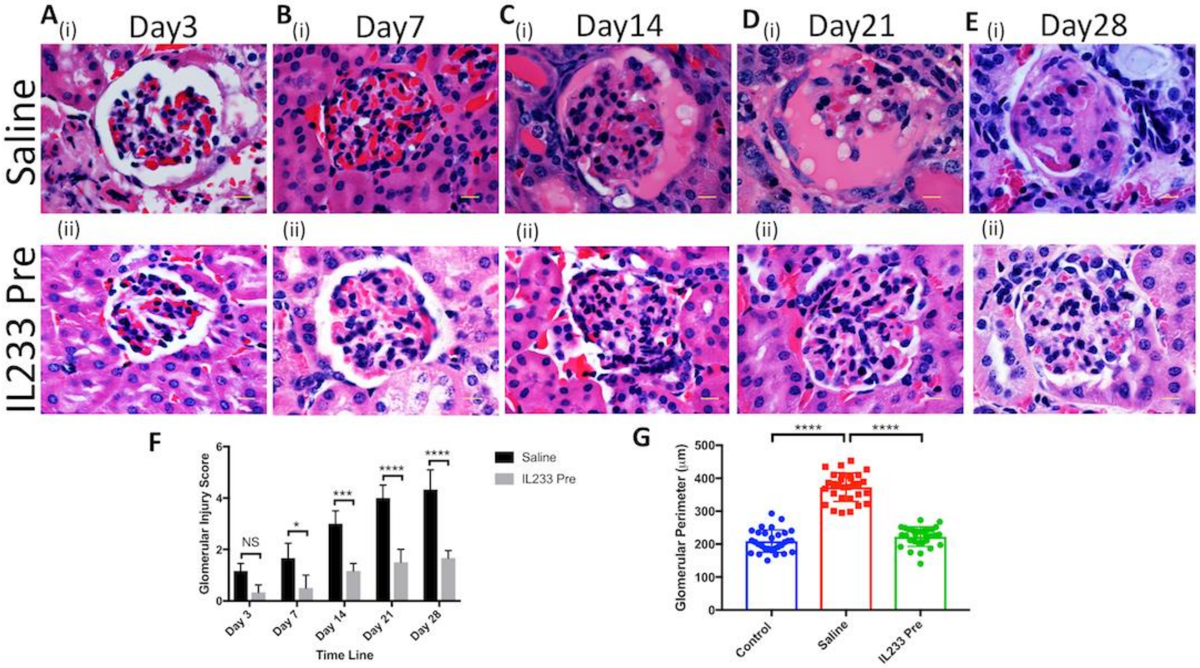

Supplement: Supplementary file 1 — Supplementary information [file 41598_2019_39886_MOESM1_ESM.pdf]
